# Mental Health Consequences of the COVID-19 Pandemic Long-Term Exposure in Italian Dermatologists

**DOI:** 10.3390/ijerph182111239

**Published:** 2021-10-26

**Authors:** Cristina Ciuluvica (Neagu), Giulio Gualdi, Marco Dal Canton, Fabrizio Fantini, Andrea Paradisi, Paolo Sbano, Marco Simonacci, Daniele Dusi, Gian Marco Vezzoni, Carmine D’Acunto, Maurizio Lombardo, Malvina Zanchi, Zucchi Alfredo, Klaus Eisendle, Francesca Prignano, Paolo Amerio

**Affiliations:** 1 Department of Medicine and Aging Sciences, University “G. d’Annunzio” of Chieti-Pescara, 66100 Chieti, Italy; giulio.gualdi@unich.it; 2 Ambulatory of Medical and Surgical Dermatology Belluno, 32100 Belluno, Italy; mdc@qderm.it; 3 Department of Dermatology, Hospital “A. Manzoni”, 23100 Lecco, Italy; f.fantini@asst-lecco.it; 4 Dermatology Unit, General Hospital “Cristo Re”, 00100 Rome, Italy; aparad78@gmail.com; 5 Dermatology Unit, General Hospital “Belcolle”, 01100 Viterbo, Italy; sbanopaolo@gmail.com; 6 Department of Dermatology, Macerata Hospital, 62100 Macerata, Italy; marcosimonacci@libero.it (M.S.); danieledusi@libero.it (D.D.); 7 “Don Bosco” Medical Center, 55049 Viareggio, Italy; gianmarcovezzoni@yahoo.com; 8 Department of Emergency, Burn Center and Dermatology, “M. Bufalini” Hospital, 47100 Cesena, Italy; carmine.dacunto@auslromagna.it; 9 Unit of Dermatological Diseases, ASST “Sette Laghi”, Hospital of “Circolo”, 21100 Varese, Italy; maurizio.lombardo@asst-settelaghi.it; 10 Ambulatory of Medical and Surgical Dermatology Venice, 30100 Venice, Italy; malvina_zanchi@yahoo.it; 11 Department of Dermatology, University of Parma, 43100 Parma, Italy; alfredozucchi57@gmail.com; 12 Clinic of Dermatology, Central Hospital of Bolzano, 39100 Bolzano, Italy; klaus.eisendle@sabes.it; 13 Department of Health Science, Section of Dermatology, University of Florence, 50121 Florence, Italy; francesca.prignano@unifi.it

**Keywords:** COVID-19, depression, anxiety, post-traumatic distress, resilience

## Abstract

The present study aimed at assessing the consequences of prolonged exposure to COVID-19 distress on mental health in non-frontline health care workers. For this purpose, we have conducted a survey on 425 Italian dermatologists, in the period February–March 2021. The psychopathological symptoms, depression, anxiety, post-traumatic stress symptoms (PTSD), as well as resilience, have been evaluated. The main factors that influence the physician’s psychological health have been also investigated. Our study showed that the physicians older than 40 years, as well as those who lived this period in company, reported more personal resources, better managing the distress. Resilience, COVID-19 beliefs, COVID-19 working difficulties, and age were the common predictors of the severe psychopathological symptoms. An interesting result is that the lower level of resilience was the most powerful predictor of a more severe depression, as well as of a higher severity of generalized anxiety disorder, but not of COVID-19 PTSD. The fear of COVID-19 was the most powerful predictor of COVID-19 PTSD. Home conditions and previous SARS-CoV2 infection constituted significant predictors of severe depressive symptoms, but not of anxiety and COVID-19 PTSD. These results are useful in a better understanding of protective and risk factors involved in COVID-19 long-term distress exposure.

## 1. Introduction

The SARS-CoV2 infection had become a pandemic in February 2020 and still, after more than a year, it imposes a great burden on health care. Italy was one of the countries most hardly hit by the pandemic, especially in the first wave, and especially in the northern part of the country. The lack of professional medical infrastructure of the health care system necessary for such a pandemic state, the paucity of knowledge on virus biology, and the lack of proper management, have generated an environment of uncertainty and fear [1,2,3,4,5]. This has impacted people’s lifestyles, causing high levels of psychological distress, anxiety, and mood alterations. Both the fear of contracting the virus and the measures adopted to counteract the spread of infection have been perceived as traumatic events [6,7]. Consequently, they can represent risk factors for many mental diseases [8], and can potentially generate important psychopathologies as depression, anxiety and post-traumatic stress disorder (PTSD) symptoms [8,9].

In this setting, health care providers had to face unprecedented challenges and workloads, as well as the fear of becoming infected and infecting their relatives. Moreover, the lockdown put in place by the Italian government limited the movements of the population, and thus, the access to the health care system. The pandemic and the subsequent lockdown had a great impact on health care providers’ psychological distress and well-being worldwide [10]. A recently published Italian survey that has been proposed to different health professionals reported that the highest psychological impact was on the doctors in the regions with the greatest number of COVID-19 cases (the northern part of the country), on females, on low job-seniority doctors, as well as on the doctors that have been directly involved in COVID-19 case management [11].

It has been reported that, in the early phase of the pandemic, frontline workers experienced significantly higher levels of mental conditions with moderate to severe symptoms, especially insomnia and anxiety, compared with the second-line workers, with depression in 50.4%, anxiety in 44.6%, and insomnia in 34.0% of health care workers [12].

In one publication performed in the midst of the pandemic, on mental distress and health involving dermatologists practicing in several countries, depression was reported by 22%, while 77% of dermatologists reported an important level of distress. The main features reported by this study were insomnia (27%) and irritability (24%) [13].

The present study aimed at assessing the consequences of prolonged exposure to COVID-19 distress as well as to investigate and describe the main factors that could influence Italian dermatologists’ psychological health, exploring whether and to what extent these factors were associated with the presence of psychopathological symptoms. We intend to identify the main risk factors that could influence mental health in a non-emergency specialty, but highly exposed to the risk of SARS-CoV2 infection, one year since the beginning of the pandemic.

In Italy, dermatology is performed by specialists in hospitals or outpatients’ clinics both privately and within the public health care system. Italian dermatologists had to face several challenges during the COVID-19 pandemic. Many were directly involved in the treatment of COVID-19 patients [14] and had to decide, among a dramatic paucity of information, on the therapeutical management of chronic inflammatory or oncologic skin diseases [15,16,17,18]. Moreover, they had to cope with the reduction in outpatient dermatological consultation and the need for new meanings of doctor to patient’s interactions such as teledermatology [14]. The rise in the number of patients with severe chronic inflammatory and oncological dermatological diseases, reported after the lockdown period, [14,15,16,17,18] has imposed an additional burden on the dermatologists’ psychological distress.

We hypothesized that the nature of dermatologic practice as well as the mental distress due to several causes associated both, to the pandemic itself and to the social and economic consequences of the imposed lockdown are important risk factors that could influence mental health among non-frontline doctors. Resilience was the personal resource investigated to better understand whether, in this context, the personality characteristics could have influenced the severe psychopathological symptomatology development.

## 2. Materials and Methods

### 2.1. Sample

Study participants were recruited from Italian dermatologists and residents in dermatology working in the north, south and, central part of Italy. The participants were asked to fill out an online self-administered structured questionnaire in the period February–March 2021. The recruitment period was chosen to set the study time approximately one year after the first peak of the COVID-19 pandemic. The survey was compiled using an online platform and took approximately 15 min to be completed. The research protocol has been approved by the Board of the Department of Medicine and Aging Sciences, Faculty of Medicine and Surgery, University G. D’Annunzio, Chieti—Pescara, Italy. All participants were informed about the privacy, ethical aspects, and data treatment. They expressed their agreement to participate in the study before starting to answer the questionnaire.

### 2.2. Instruments

The Demographic Data, Working, and Personal COVID-19 Survey has been used to collect information related to demographic data, working, and personal COVID-19 context. The psychopathological symptoms have been investigated using Patient Health Questionnaire (PHQ-9) for depression [19,20], Generalized Anxiety Disorder Scale (GAD-7) for anxiety [21,22,23], Post-Traumatic Stress Disorder Related to COVID-19 Questionnaire (COVID-19-PTSD) for post-traumatic stress symptoms [24,25], Resilience Scale (RS) for resilience [26,27]. The Fear of COVID-19 Questionnaire was adapted from previously published materials [7] to evaluate the fear of SARS-CoV2 contagion in long-term exposure. The COVID-19 Risk Factors Questionnaire (ad hoc Questionnaire) was created for the COVID-19 emergency and referred to the perception of the main COVID-19 working risk factors. All data were categorized into four groups of variables: Sociodemographic, COVID-19 working experience, COVID-19 personal experience as well as personal resources.

The Demographic Data, Working and, Personal COVID-19 Survey is a questionnaire organized in three parts to collect information related to demographic data, working and, personal COVID-19 context. The first section of the survey collected information on demographic variables, including gender (men or women), age, civil status, education, and pre-existing medical conditions. The second section included questions to investigate the personal context related to the COVID-19 pandemic: home conditions, SARS-CoV2 previous infection, SARS-CoV2 infection of at least one family member or friend. The third part contained items describing working context: region, workplace setting, working position.

Patient Health Questionnaire (PHQ-9) is a 9-item self-report measure designed to screen for depression in primary care and other clinical settings [19]. The PHQ-9 items assess the presence of sadness, emptiness, or guilt, accompanied by somatic and cognitive changes (e.g., low energy, sleeping trouble, concentration difficulties) that significantly affect the individual’s capacity to function. Subjects were asked to report the presence of each symptom during the last two weeks on a 4-point rating scale from 0 (“not at all”) to 3 (“nearly every day”). The total PHQ-9 scores range from 0 to 27, scores of <5 represent the absence of depression symptoms, and higher scores indicating greater severity of depression. The PHQ-9 is widely used in clinical and research settings and be provided with sound psychometric characteristics [20]. Within this sample, Cronbach’s α was 0.82.

Generalized Anxiety Disorder Scale (GAD-7) is a 7-item self-report questionnaire that is widely used in clinical and research settings for screening anxiety [21]. Anxiety symptoms include, for example, excessive fear, feeling nervous, trouble relaxing, and anticipation of future threats. Participants were asked to rate how often they have been bothered by each symptom during the past two weeks. Responses are scored on a 4-point rating scale from 0 (“not at all”) to 3 (“every day”). Total scores range from 0 to 21, scores of <5 represent the absence of anxiety symptoms, and higher scores reflecting higher severity levels of generalized anxiety disorder symptomology. The GAD-7 has good reliability, construct, factorial, and procedural validity [22,23]. Within this sample, Cronbach’s α was 0.89.

Post-Traumatic Stress Disorder Related to COVID-19 Questionnaire (COVID-19-PTSD) is a self-report measure created to assess specific symptoms concerning the risk of PTSD in the COVID-19 pandemic. PTSD is a psychiatric disorder caused by a terrifying event, perceived as a trauma, which affects directly or indirectly the individual (e.g., severe accident or injury, threat to physical safety, death or threat of death, sexual assault, natural disasters, war, etc.) [24]. All the questions refer to the previous seven days during the COVID-19 pandemic. The questionnaire includes 19 items, requiring a response on a 5-point Likert scale, from 0 (not at all) to 4 (extremely). The instructions provided to the respondents are: “Referring to the current situation, characterized by the COVID-19 pandemic and the social measures implemented to contain it, indicate how you feel for each of the following dimensions”. The questionnaire is organized into seven clusters: intrusion, avoidance, negative affect, anhedonia, dysphoric arousal, anxious arousal, and externalizing behavior. Within this sample, internal consistency was good for all subscales, Cronbach’s α ranging between 0.78 and 0.86.

Resilience Scale (RS) is one of the most used scales to measure dispositional resilience in adults. Resilience is defined as “a personal trait that moderates negative effects of stress and promotes adaptation “. Resilience is, therefore, considered by the makers of the ladder to be an innate characteristic, present in each person albeit to a different extent and that can be strengthened based on how life events are faced and overcome [26]. There are two versions of the scale that differed in items number: 25 items version and 10 items version, but both demonstrated a good fit and clinical utility. The factorial analysis conducted on the version consisting of 25 items showed the existence of two factors, while the analysis on the 10 items version demonstrated a single-factorial structure. In our research, we used the 10 items version of the scale [26,27]. The RS is a seven-point Likert scale with response options ranging from 0 (strongly disagree) to 6 (strongly agree). Within this sample, Cronbach’s α was 0.82.

Fear for COVID-19 Questionnaire is an 8-item scale that evaluates the fear for COVID-19 contagion [7]. The questionnaire was initially constructed to evaluate the fear of contagion during the COVID-19 lockdown. We adapted it to evaluate the fear of contagion during the COVID-19 pandemic. The instructions provided to the participants are: “Referring to the current situation, characterized by the COVID-19 pandemic, indicate how you thought for each of the following dimensions in the last months”. Participants answered on a scale from 0 (not at all) to 4 (extremely). In our sample (*n* = 425), the component structure and reliability of the questionnaire were explored using principal component analysis (PCA) and Cronbach’s α. The oblique (Promax) rotation was used. The results from these analyses revealed two factors moderately correlated (r > 0.40). The first factor, “Beliefs on COVID-19 contagion” (COVID-19 Beliefs), composed of four items showing satisfactory loadings (i.e., >0.41) reflects the conviction of being infected, either in the past or in the future. The second factor, “Consequences of COVID-19 contagion” (COVID-19 Consequences), included the other four items showing also satisfactory loadings (i.e., >0.40) reflects the possibility of suffering severe consequences due to the contagion (i.e., to be hospitalized or to die). The second factor included one item that saturate below 0.70 but presenting a value very close to 0.70 (0.68) was not excluded from the cluster structure. (Table 1) The scores ranging from 0 to 16 were computed by averaging the constituent items of each scale. Higher scores reflect a major fear of COVID-19 contagion. Cronbach’s α showed good values for both the “Beliefs on COVID-19 contagion”, α = 0.79, and “Consequences of COVID-19 contagion” scale, α = 0.72.

COVID-19 Risk Factors Questionnaire (ad hoc Questionnaire) is a 6-item questionnaire created for the COVID-19 emergency and referred to the perception of the main COVID-19 working risk factors. The items presented in Table 2 were constructed to evaluate two categories of factors that can lead to mental risk: emotional and working difficulties. Participants answered on a Likert scale from 0 (not at all) to 4 (extremely). The component structure and reliability of the questionnaire were explored in our sample (*n* = 425), using principal component analysis (PCA) and Cronbach’s α. The oblique (Promax) rotation was used. The results revealed two factors moderately correlated (r > 0.42) each factor containing three items. Within the two factors, the items showed satisfactory loading (i.e., >0.42). The first factor, “COVID-19 Emotional Working Risk”, reflects the emotional perceived risk due to COVID-19 long time working exposure. Specifically, the questions evaluate the extent to which emotions such as the fear of being infected and transmitting the infection to loved ones; the suffering caused by the loss of patients, or the separation from the family are perceived as risk factors. The second factor, “COVID-19 Working Difficulties”, reflects the perception of the work difficulties due to COVID-19 presence. The questions evaluate the extent to which the fatigue due to prolonged exposure, the communication difficulties with the patients, and the work procedures changes are perceived as risk factors. The scores of each scale, ranging from 0 to 15, were computed by averaging the responses to the constituent items. Higher scores reflect a major COVID-19 working risk perception. Internal consistency was tested with Cronbach’s α. The results showed good values for both the COVID-19 Emotional working risk, α = 0.73, and COVID-19 Working difficulties scale, α = 0.77.

### 2.3. Statistics

Descriptive statistics were generated for all the study variables listed above: sociodemographic (age, civil status, location, home conditions), medical factors (pre-existing health conditions), COVID-19 factors (working positions, workplace setting, previous infection with SARS-CoV2, SARS-CoV2 infection of at least one family member or friend, COVID-19 psychological compo-nents (beliefs on COVID-19 contagion, consequences of COVID-19 contagion, COVID-19 emotional working difficulties, COVID-19 working difficulties), clinical variables (depression, anxiety, COVID-19 PTSD, intrusion, avoidance, negative affect, anhedonia, dysphoric arousal, anxious arousal, external behavior), and personality characteristics (resilience). The continuous variables have been checked by the distribution using the skewness and the kurtosis values. Student’s *t*-test was used to compare clinical variables, personality characteristics, COVID-19 psychological factors for between-group differences considering gender, age, civil status, pre-existent medical conditions, home conditions, region, working position, workplace setting, previous COVID-19 infection, one’s relatives’ COVID-19 infections. The standardized mean difference was used as a measure of effect size (Cohen’s d). A Cohen’s d of 0.20–0.50 is considered small, 0.50–0.80 moderate, and >0.80 large. Analysis of variance (ANOVA) was used for evaluating psychological and clinical variables for between-group comparison considering the geographic location (North, Centre, and South of Italy). The hierarchical moderated linear regression analysis was used to determine the best predictors for the three main clinical variables of the study. Three separate regression models having three different dependent variables were analyzed: Model A—Depression, Model B—Anxiety, and Model C—COVID-19 PTSD. The independent variables used as predictors for the three models were: sociodemographic, COVID-19 factors, COVID-19 psychological components, and personality characteristics. To perform the regression analysis, the categorical variables were dummy coded. For this purpose, we grouped the independent variables in four classes: (1) sociodemographic: gender, age, civil status, and pre-existing health conditions (2) COVID-19 individual context: home conditions, living in a COVID-19 highly impacted region, previous infection with SARS-CoV2, SARS-CoV2 infection of at least one family member or friend, fear for COVID-19 (COVID-19 belief, COVID-19 consequences) (3) COVID-19 working context: working position, workplace setting, COVID-19 working emotional difficulties, COVID-19 working difficulties (4) personal resources: resilience.

The independent variables were entered in the regression analysis in four steps: Step 1—sociodemographic data; Step 2—COVID-19 individual context; Step 3—COVID-19 working related factors; Step 4—personal resources.

## 3. Results

### 3.1. Descriptive Analysis of Sociodemographic, Workload and Clinical Characteristics

The sociodemographic and workload characteristics of the sample are presented in Table 3. Four hundred and twenty-five physicians completed the online survey. The mean age was 43.3 (SD = 12.52), the age range between 25 and 73 years old, 243 were women (56.9% of the sample). The regions distribution of the sample showed higher participation in the study of the northern part of the country (221; 52%). The sample was homogeneous according to gender (55.9% women) and age (45.2% <40 years). Dermatologists directly involved in the COVID-19 patients’ care were 30.4% of the total sample while those who experienced at least one patient loss due to COVID-19 were 21.3%. Collected data showed that most of the participants were working as hospital staff (57.3%). The number of dermatologists working in COVID-19 highly impacted regions (North of Italy), was higher than those located in COVID-19 lower impacted regions (Centre and South); 52.0%, 22.8% and 27.2%, respectively.

Two-hundred ninety-nine (70.6%) dermatologists had a relative infected by SARS-CoV2 at any time while only forty-seven (15%) experienced personally COVID-19.

For depression, the mean PHQ-9 score was 5.66, SD = 4.71 (women 6.32, SD = 4.60; men 4.79, SD = 4.73), for anxiety, the mean of GAD-7 score was 4.41, SD = 4.33 (women 5.10, SD = 4.43; men 3.50, SD = 4.04), while for COVID-19 PTSD symptoms, the mean COVID-19 PTSD score was 20.26, SD = 14.20 (women 22.50, SD = 15.81; men 17.29, SD = 13.06). Two hundred and twenty-eight (54%) of the participants suffered from severe depressive symptoms (PHQ-9 score > 5). Instead, 176 dermatologists (42%) showed severe generalized anxiety disorder symptomatology (GAD-7 score > 5). One-hundred and thirteen physicians (27%) presented with severe PTSD symptomatology.

### 3.2. The Relation between the Participants’ Characteristics, the Clinical and the Psychological Variables

The differences between groups in clinical variables (depression, anxiety, COVID-19 PTSD, and its components: intrusion, avoidance, negative affect, anhedonia, dysphoric arousal, anxious arousal, external behaviors) are presented in Table 4. Gender, age, and home conditions have been demonstrated as the main factors that generated important differences in clinical variables. Long-term exposure to COVID-19 distress psychological impact was higher in women than in men, as well as in younger than in older physicians. Women scored higher in eight out of ten clinical variables. (Table 4) Effect sizes indicate that females reported higher depression (d = 0.56) and higher anxiety (d = 0.53) than men. Women experience also higher levels of COVID-19 PTSD (d = 0.57) in some of its components: intrusion (d = 0.48), avoidance (d = 0.37), negative affect (d = 0.43), anhedonia (d = 0.43), and anxious arousal (d = 0.58) compared to men. Younger physicians reported higher levels of depressive and anxiety symptoms, d = 0.35 and d = 0.52, respectively, as well as COVID-19 PTSD (d = 0.36), in some of its components: negative affect (d = 0.35), anhedonia (d = 0.51), dysphoric arousal (d = 0.24), and external behavior (d = 0.26) (Table 4).

Dermatologists living alone during the pandemic reported higher depressive symptoms (d = 0.61) and anxiety (d = 0.46) compared to those who shared the house with someone (family, relatives, friends). Living alone also leads to higher scores in COVID-19 PTSD symptoms (d = 0.45), in some of its components: anhedonia (d = 0.513), external behavior (d = 0.53), and negative affect (d = 0.26) (Table 4).

Higher depressive symptoms (d = 0.48), as well as two COVID-19 PTSD mechanisms (dysphoric arousal, d = 0.34, and external behaviors, d = 0.32), have been reported by physicians who suffered a previous SARS-CoV2 infection (Table 4).

Women demonstrated a higher perception of COVID-19 emotional risk (d = 0.51), as well as a higher perception of COVID-19 working difficulties (d = 0.34), and stronger beliefs regarding COVID-19 contagion. The fear of consequences due to onstrated a higher perception of COVID-19 e contagion resulted higher in older participants (d = 0.42) as well as in the participants who suffered a previous chronic disease (d = 0.34) (Table 3).

### 3.3. The Relation between Workload Characteristics, the Clinical and the Psychological Variables

Working directly with COVID-19 patients, working in a hospital, living in a COVID-19 higher impacted region, experiencing SARS-CoV2 relatives’ infection, or having a pre-existent medical condition did not affect the psychological health of Italian dermatologists. Instead, these latter factors influenced, to a smaller degree, the COVID-19 psychological variables: fear of COVID-19 (COVID-19 beliefs and COVID-19 consequences) as well as the perceived working risk (COVID-19 emotional working risk, COVID-19 working difficulties) (Table 5) At the same time, dermatologists who worked in an out-patient office (d = 0.30) and those who worked in the southern region of Italy (*p* < 0.05) reported higher perceived difficulties in their work due to COVID-19 induced changes in working procedures. The physicians directly involved in the care of COVID-19 patients perceived higher emotional difficulties (d = 0.27). (Table 5)

### 3.4. Prediction Models for Clinical Variables: Depression, Anxiety, and COVID-19 PTSD

The three regression models constructed to predict depression, anxiety, and COVID-19 PTSD showed significant changes for each class of variables added in the models. The results including the significant coefficients of change in the four regression steps are summarized in Table 6. The final moderated linear regression models are presented in Table 7.

The model A (*F* (15) = 23.307, *p* ≤ 0.001) accounted for approximately 46% of the variance in depressive symptoms (*R^2^* = 0.462 (*Adj R*^2^ = 0.443), *F*-change = 112.479, *p* < 0.001). Resilience (*β* = −0.41, *p* < 0.001), beliefs on COVID-19 contagion (*β* = 0.21, *p* < 0.001), COVID-19 work difficulties (*β* = 0.16, *p* < 0.001), previous infection with SARS-CoV2 (β = −0.14, *p* < 0.001), home conditions (*β* = −0.13, *p* = 0.001), age (*β* = −0.10, *p* < 0.05), and gender (*β* = 0.09, *p* < 0.05) were found to be significant predictors of depressive symptoms.

The model B (*F* (15) = 17.934, *p* ≤ 0.001)) accounted for approximately 40% of the variance in anxiety (*R*^2^ = 0.402 (*Adj R*^2^ = 0.376), *F*-change = 75.758, *p* < 0.001). Resilience (*β* = −0.35, *p* < 0.001), beliefs on COVID-19 contagion (*β* = 0.19, *p* < 0.001), age (*β* = −0.17, *p* < 0.001), COVID-19 working difficulties (*β* = 0.14, *p* < 0.01), consequences of COVID-19 contagion (*β* = 0.10, *p* < 0.05), and gender (*β* = 0.09, *p* < 0.05) were found to be significant predictors of anxiety.

The model C (*F* (15) = 24.484, *p* ≤ 0.001)) accounted for approximately 47% of the variance in COVID-19 PTSD (*R*^2^ = 0.474 (*Adj R*^2^ = 0.445), *F*-change = 44.971, *p* < 0.001). Beliefs on COVID-19 contagion (*β* = 0.28, *p* < 0.001), resilience (*β* = −0.25, *p* < 0.001), COVID-19 working difficulties (*β* = 0.23, *p* < 0.001), age (*β* = −0.17, *p* < 0.001), and consequences of COVID-19 contagion (*β* = 0.16, *p* < 0.001) were found to be significant predictors of COVID-19 PTSD.

## 4. Discussions

The present study explored the impact of long-term COVID-19 pandemic exposure on mental health status in non-frontline health care workers, assessing depression, anxiety, COVID-19 PTSD, and the most important psychological protection mechanism, resilience, in a large sample of dermatologists (*n* = 425).

The present study explored the impact of long-term COVID-19 pandemic exposure on mental health status in non-frontline health care workers, assessing depression, anxiety, COVID-19 PTSD, and an important psychological protection mechanism, resilience, in a large sample of dermatologists (*n* = 425).

The clinical variables have been assessed in relation to four categories of potential influence factors: sociodemographic; individual context; working related and personal resources.

### 4.1. The Impact of the Long-Term Exposure of COVID-19 Distress on the Psychopathological Symptomatology

Although this physicians’ category was not directly involved in COVID-19 patients’ management, the proportion of specialists that reported severe depressive symptoms was very high (54%). Anxiety disorder symptomatology was also reported in a great portion of the sample (42%), while 27% reported higher levels of post-traumatic stress. Unexpectedly, this indicates that one year of COVID-19 imposed changes had lifted a great toll on the mental health of dermatologists. These findings are worse than those reported by a single study on dermatologists performed in the mists of the pandemic [13].

In studies that reported the distress year following the outbreak, the perceived stress was higher amongst exposed health care workers vs. the non-exposed ones, and had increased overtime [28]. In addition, a year following the outbreak, the perceived stress was higher amongst health care workers, compared to non-health care workers, increasing over time only for health care workers [28].

In line with this evidence, our results on higher levels of depression and anxiety demonstrate that the impact on dermatologists’ mental distress increased along with the time the professionals were exposed to the consequences of the pandemic. Distress was associated with a health care system that was not adequately prepared (scarcity of personal protective equipment), with financial uncertainties caused by the lockdown, with a rural location of practice, with the use of teleconsultation, and with dermatologists’ younger age [26].

### 4.2. The Influence of the Participant’s Characteristics on the Psychopathological Symptomatology

We were able to demonstrate that long-term COVID-19 pandemic consequences have also impacted heavily on younger dermatologists that were more stressed one year after the beginning of the pandemic. To explain this evidence, we can assume that younger physicians could present with poor coping ability, due to inexperience and lesser professional skills, compared to an experienced dermatologist. Younger dermatologists seem to suffer more than seniors relating to the change in the workload and the perceived risk of infection, thus resulting in a higher incidence of psychopathological symptoms.

Women experienced more severe depressive and anxious symptoms than men. Moreover, as expected from the evidence of the literature regarding the general population in the pandemic period [29], both women and younger subjects reported higher post-traumatic symptomatology. They presented as predominant mechanisms of dysfunctional distress: anxious arousal, intrusion, negative affect, avoidance, and anhedonia. This has been confirmed also for other health care workers during the pandemic [30]. Our study revealed that physicians that lived alone during this extremely difficult period showed more severe clinical symptomatology. We may presume that for this professional health care category living with friends or relatives had a protective role in preventing psychological distress during the pandemic. This result is supported by the evidence that depression and anxiety have been related to loneliness during the lockdown and implementation of social distancing measures [31,32,33]. Our report of a critical level of PTSD symptoms in 27% of the dermatologists after long-term COVID-19 pandemic impact is strikingly higher than that reported in patients with mild COVID-19 (17.3%) [34]. Several papers on health care workers showed that PTSD incidence during the pandemic ranged between 9 and 49.38% [30]. However, PTSD higher scores were reported by emergency personnel, our results were far higher than the mean reported PTSD levels of non-frontline workers, probably due to the longer time of exposure to COVID-related stressors [30]. The main predictors of PTSD reported in the literature were: young age, low work experience, female gender, heavy workload, working in unsafe settings, working in frontline, and lack of training and social support [21]. Our results fit perfectly into these characteristics and demonstrated that, even one year after the start of the lockdown measures, more than one-quarter of dermatologists (a non-frontline health care workers category) still show critical levels of PTSD symptomatology. At the same time, women and younger physicians reported major dysfunctional distress mechanisms as anxious arousal, intrusion, negative affect, avoidance, anhedonia.

In our sample, dermatologists who suffered a previous infection with SARS-CoV2 reported critical levels of depressive disorder symptomatology. It is still unclear whether COVID-19 can induce psychiatric symptoms during or after the acute illness phase [24] so we can only speculate on the fact that having had a previous infection could be depression’s cause or effect.

### 4.3. The Influence of the Workload Characteristics on the Clinical and Psychological Variables

The data collected by our customized questionnaires showed that women perceived a higher working risk due to COVID-19 exposure and higher fear of COVID-19 compared to men. Women showed a major emotional impact due to the fear of being infected and transmitting the virus to their own families as well as for the suffering from the loss of patients and colleagues due to COVID-19.

Indeed, in the literature female gender was reported together with the perception of a high risk of contracting COVID-19 to be positively correlated with higher psychological distress [25].

We were also able to demonstrate that women perceived major difficulties in managing the changes in work procedures, in communication and relationships with patients as well as in managing the physical fatigue related to working hours and the use of protective devices.

This class of difficulties was perceived as major also by the physicians working in a territorial structure as well as of those working in the southern part of Italy. These results of our study stemming from a sample evaluated after a long-time stress exposure are very close to those reported in an earlier published survey on worldwide dermatologists [26].

Older physicians and those who suffered a previous SARS-CoV2 infection reported major fear regarding the contagion consequences. This may be explained by the fact that physicians could have been more aware of the earlier evidence that the older population presented with a major risk of severe SARS-CoV2 infection disease and complications.

Dermatologists working in contact with infected patients reported higher emotional working risk showing major fear regarding the probability of being infected themselves or infecting their loved ones. However, the direct involvement of dermatologists in COVID-19 patients’ management did not determine any significant difference in terms of depression, anxiety, or PTSD symptomatology compared to those not directly involved. Nonetheless, both groups reported critical levels of depression.

### 4.4. The Influence of the Personal Resources on the Psychopathological Symptomatology

Resilience was an important variable in our study, reflecting the existence of important personal resources, as well as a changeable factor that could protect against severe psychological impairment [35,36,37]. As expected, high resilience values appear as a strong protective factor concerning the risk of developing severe psychopathologies. The pandemic impact, social distancing regulations, and changes in daily work activity acted as stressors.

Our study showed that the physicians older than 40 years, as well as those who were able to develop important relationships and were living this period in company, in the context of prolonged exposure to COVID-19 as a powerful stressor, reported more personal resources, better distress management, and higher resilience. Older dermatologists may have more experience with stressors, so they showed more resilience to stress.

Through the three regression models, we found that resilience, COVID-19 belief, COVID-19 working difficulties, and age were the common predictors of the severe psychopathological symptoms in physicians after one year of exposure to COVID-19 pandemic consequences.

An interesting result of our study is that the lower level of resilience was the most powerful predictor of greater severity of depression, as well as of greater severity of generalized anxiety disorder, but not of COVID-19 PTSD. The fear of COVID-19 (COVID-19 belief) resulted to be the most powerful predictor of COVID-19 PTSD. The powerful contribution of resilience as protective factor in COVID-19 long-term distress suggested by the results of our study is in line with the literature. Previous studies showed that resilience is a protective factor for PTSD and depression [35,36,37].

Moreover, home conditions and previous SARS-CoV2 infection have been significant predictors of severe depressive symptoms, but not of generalized anxiety and COVID-19 PTSD symptoms. Gender was a lower predictor in depressive and anxious symptomatology, but not for COVID-19 PTSD.

### 4.5. Limits and Prospective

Even if the results of the present study are interesting in offering new data regarding the psychopathological symptomatology related to COVID-19 long-term context, the limits of our research need to be taken into consideration. On one hand, we acknowledge that our work may suffer from the bias as being based on online self-reported experiences of dermatologists, and on the other hand, that the number of physicians who were aware of the survey and decided to participate in the study represents approximatively 15% of the total number of Italian dermatologists. Consequently, these results are useful in a better understanding of the psychopathological risk of long-term exposure of COVID-19 distress in Italian dermatologists, and are not enough to provide a universally valuable psychopathological profile of all Italian dermatologists. Future studies could explore the same issues in different groups of health care professionals highlighting the differences between the different categories of health care workers.

## 5. Conclusions

Contrary to most of the studies published on psychopathological symptomatology in health care workers that have been related to the early period of the pandemic, as well as in the professionals directly involved in COVID-19 management, in this work, we reported the impact of psychopathological symptomatology in non-frontline health care workers category (dermatologists), after one year of the pandemic. COVID-19 pandemic, social distancing, and changes in daily work practices may cause a profound threat to psychological health also for this health care workers category.

Nevertheless, we showed that, after one year of COVID-19 pandemic consequences, even dermatologists, although being non-frontline workers, have been widely exposed to psychopathological symptoms such as: depression, anxiety, and PTSD.

Many factors were associated with a higher risk of developing this symptomatology; however, resilience was reported to be the most important protective factor. Since resilience has been suggested to be a modifiable factor, the early detection of those at risk of more serious mental impairment would be particularly important, in the context of protracted stress, as well as having implications for earlier intervention opportunities.

These results are useful in a better understanding of protective and risk factors involved in COVID-19 long-term distress exposure. Prevention, as well as emergency psychological interventions, aimed at reducing COVID-19 fear and enhancing resilience, may help to reduce the risk to develop important psychopathological symptomatology in health care workers.

## Figures and Tables

**Table 1:** Pattern matrix of the PCA for the Fear of COVID-19 questionnaire.

	Factors	Factor Loading
	First Factor/Beliefs on COVID-19 Contagion	
1.	I often thought I was infected with the virus.	0.724
2.	I think I could be infected with the virus in the future.	0.804
3.	I think that a dear or close person to me could potentially be infected with the virus.	0.709
4.	I think that a dear or close person to me could potentially be infected with the virus in the future.	0.813
	Second Factor/Consequences of COVID-19 contagion	
5.	I think that a person infected with the virus could recover.	0.741
6.	I think that a person infected with the virus could die.	0.730
7.	I think it is probable that I would recover after being infected with the virus	0.702
8.	I think that being infected with the virus could be lethal for me.	0.687

**Table 2:** Pattern matrix of the PCA for the COVID-19 Risk Factors Questionnaire.

	Factors	Factor Loading
	First Factor/COVID-19 Emotional Working Risk	
1.	The fear of getting the infection and infecting your loved ones	0.734
2.	Separation often prolonged by one’s family	0.710
3.	Suffering from the loss of patients and colleagues	0.742
	Second Factor/COVID-19 Working Difficulties	
4.	Changes in work procedures and relationships with patients	0.791
5.	Physical fatigue related to working hours and the use of protective devices	0.712
6.	Communication difficulties and the need to provide greater emotional support to patients with COVID-19	0.701

**Table 3:** The sociodemographic and workload characteristics of the sample.

Total Sample; *n* (%)	425 (100%)
Gender; *n* (%)	
Men	183 (43.1%)
Women	243 (56.9%)
Age	
<40; *n* (%)	192 (45.2%)
≥40; *n* (%)	133 (54.8%)
Men’s Age; Mean (SD)	46.02 (14.28)
Women’s Age; Mean (SD)	41.24 (10.58)
Civil Status; *n* (%)	
Unmarried/Widower	148 (34.8%)
Married/Cohabitant	249 (58.5%)
Separate/Divorced	28 (6.6%)
Preexisting Medical Conditions; *n* (%)	
None	327 (76.9%)
Cardiac	14 (3.2%)
Respiratory	16 (3.9%)
Dermatologic	4 (0.9%)
Oncologic	10 (2.3%)
Psychiatric	3 (0.7%)
Other Diseases	56 (13%)
Italy Regions; *n* (%)	
North	221 (52%)
Center	97 (22.8)
South	106 (24.9)
Home Conditions; *n* (%)	
Alone	117 (28.2%)
Together with family, close friends	308 (72.4%)
Workplace; *n* (%)	
University Hospital	48 (11.3%)
Clinical Hospital	111 (26.1%)
Public Health Territorial Office	26 (6.15%)
Private Health Office	117 (26.5%)
Combined	123 (28.3%)
COVID-19 Working Positions; *n* (%)	
Directly involved in the COVID-19 patients care	173 (40.7%)
Directly involved in the COVID-19 patients care that lose their lives	94 (21.3%)
Directly involved in the COVID-19 patients care in IT	11 (2.4%)
Not Directly involved in the COVID-19 patients care	252 (59.3%)
Previously Infected with SARS-CoV2; *n* (%)	
Yes	47 (15%)
No	378 (88.9%)
Family Member or Friend Infected with SARS-CoV2; *n* (%)	
Yes	299 (70.6%)
No	126 (29.6%)

**Table 4:** Differences between Groups (Student’s *t*-Test, ANOVA One Way). Clinical Variables: Depression; Anxiety; COVID-19—PTSD. COVID-19—PTSD Mechanisms measured as subscales of COVID-19 PTSD Questionnaire: Intrusion, Avoidance, Anhedonia, Dysphoric Arousal, Anxious Arousal, External Behavior.

Groups	Depression Mean (SD)	Anxiety Mean (SD)	COVID-19-PTSD Mean (SD)	Intrusion Mean (SD)	Avoidance Mean (SD)	Negative Affect Mean (SD)	Anhedonia Mean (SD)	Dysphoric Arousal Mean (SD)	Anxious Arousal Mean (SD)	External Behavior Mean (SD)
Total Sample	5.66 (4.71)	4.41 (4.33)	20.26 (14.20)	4.34 (3.75)	2.49 (1.89)	2.61 (2.63)	3.70 (2.85)	1.72 (1.72)	2.66 (1.99)	2.74 (3.03)
Gender										
Men	4.79 (4.73)	3.50 (4.04)	17.29 (13.06)	3.57 (3.35)	2.19 (1.80)	2.06 (2.30)	3.17 (2.64)	1.62 (1.52)	2.13 (1.69)	2.55 (2.87)
Women	6.32 (4.60)	5.10 (4.43)	22.50 (15.81)	4.92 (3.93)	2.71 (1.93)	3.03 (2.79)	4.10 (2.95)	1.79 (1.85)	3.06 (2.10)	2.89 (3.14)
*t*	−3.355	−3.866	−3.715	−3.820	−2.846	−3.913	−3.428	−1.014	−5.019	−1.166
*p*	0.001 **	0.000 ***	0.000 ***	0.000 ***	0.005 **	0.000 ***	0.001 **	0.311	0.000 ***	0.244
d	0.567	0.538	577	0.482	0.377	0.439	0.432	0.101	0.587	0.111
Age										
<40	6.30 (4.65)	5.17 (4.35)	22.44 (15.50)	4.58 (3.85)	2.57 (1.92)	3.12 (2.73)	4.34 (2.94)	1.95 (1.81)	2.82 (2.08)	3.06 (3.10)
≥40	5.13 (4.71)	3.79 (4.22)	18.46 (14.17)	4.14 (3.66)	2.42 (1.87)	2.19 (2.47)	3.18 (2.68)	1.53 (1.61)	2.53 (1.90)	2.48 (2.95)
*t*	2.552	3.319	2.760	1.220	0.795	3.660	4.257	2.519	1.494	1.989
*p*	0.011 *	0.001 **	0.006 **	0.223	0.427	0.000 ***	0.000 ***	0.012 *	0.136	0.047 *
d	0.351	0.522	0.367	0.117	0.078	0.357	0.513	0.245	0.145	0.264
Workplace Setting										
Hospital	5.67 (4.57)	4.33 (4.31)	19.65 (14.54)	4.13 (3.74)	2.35 (1.85)	2.52 (2.49)	3.76 (2.78)	1.74 (1.72)	2.53 (1.88)	2.61 (2.96)
Ambulatorial	5.64 (4.91)	4.52 (4.38)	21.09 (15.38)	4.62 (3.76)	2.68 (1.94)	2.74 (2.82)	3.62 (2.96)	1.68 (1.72)	2.83 (2.12)	2.92 (3.12)
*t*	0.061	−0.436	−0.989	−1.319	−1.755	−0.876	0.498	0.368	−1.534	−1.047
*p*	0.951	0.663	0.323	0.188	0.080	0.382	0.618	0.713	0.126	0.296
d	0.007	0.043	0.097	0.130	0.174	0.083	0.048	0.034	0.150	0.115
COVID-19 Working Positions										
Directly involved	6.05 (4.73)	4.43 (4.12)	21.55 (15.06)	4.67 (3.67)	2.64 (1.91)	2.78 (2.59)	3.75 (2.82)	1.95 (1.81)	2.68 (2.05)	3.08 (3.17)
Not directly involved	5.51 (4.70)	4.42 (4.43)	19.75 (14.82)	4.20 (3.74)	2.43 (1.88)	2.55 (2.65)	3.68 (2.88)	1.62 (1.67)	2.65 (1.96)	2.60 (2.96)
*t*	1.088	.039	1.148	1.189	1.025	0.820	0.224	10.839	0.133	1.482
*p*	0.277	0.969	0.252	0.235	0.306	0.413	0.823	0.067	0.895	0.139
d	0.114	0.002	0.120	0.126	0.110	0.087	0.024	0.191	0.015	0.158
Previous Infection with SARS-CoV2										
Yes	7.83 (5.63)	5.17 (4.45)	22.06 (15.20)	4.77 (4.04)	2.70 (2.10)	2.72 (2.45)	3.55 (2.88)	2.17 (1.68)	2.60 (2.21)	3.55 (3.25)
No	5.39 (4.52)	4.32 (4.31)	20.03 (14.87)	4.29 (3.71)	2.46 (1.87)	2.60 (2.66)	3.72 (2.85)	1.66 (1.71)	2.67 (1.96)	2.64 (2.99)
*t*	3.388	1.272	0.866	0.827	0.824	0.327	−0.346	1.948	−0.230	1.952
*p*	0.001 **	0.204	0.390	409	0.411	0.745	0.707	0.056 *	0.818	0.052 *
d	0.479	0.171	0.135	0.123	0.121	0.047	0.059	0.339	0.034	0.321
SARS-CoV2 infection of at least one Family Member or Friend										
Yes	5.55 (4.62)	4.41 (4.25)	20.78 (15.05)	4.55 (3.83)	2.58 (1.96)	2.74 (2.65)	3.68 (2.78)	1.69 (1.71)	2.73 (1.97)	2.81 (3.04)
No	5.92 (4.93)	4.42 (4.55)	19.02 (14.53)	3.83 (3.50)	2.26 (1.71)	2.31 (2.58)	3.76 (3.03)	1.79 (1.73)	2.49 (2.02)	2.59 (3.02)
*t*	−0.743	−0.027	1.123	1.874	1.683	1.554	−0.274	−0.529	1.174	0.680
*p*	0.458	0.978	0.263	0.062	0.094	0.121	0.784	0.597	0.241	0.497
d	0.077	0.002	0.120	0.196	0.173	0.164	0.027	0.058	0.125	0.072
Preexisting medical conditions										
Yes	6.02 (5.25)	4.79 (5.11)	20.15 (16.15)	4.41 (4.19)	2.54 (1.95)	2.45 (2.78)	3.48 (2.82)	1.63 (1.65)	2.80 (2.18)	2.85 (3.33)
No	5.55 (4.54)	4.30 (4.07)	20.29 (14.53)	4.32 (3.61)	2.47 (1.88)	2.66 (2.59)	3.77 (2.86)	1.74 (1.74)	2.62 (1.93)	2.71 (2.94)
*t*	−0.867	−0.973	0.078	−0.208	−0.319	0.697	0.882	0.573	−0.776	−0.393
*p*	0.387	0.331	0.938	0.835	0.750	0.486	0.379	0.567	0.438	0.695
d	0.096	0.106	0.009	0.022	0.035	0.078	0.102	0.065	0.087	0.058
Italy Regions										
North	5.71 (4.72)	4.42 (4.32)	20.05 (14.99)	3.87 (3.83)	2.41 (1.89)	2.64 (2.67)	2.77 (2.89)	1.61 (1.63)	2.54 (2.07)	2.69 (2.94)
Center	5.53 (4.94)	4.51 (4.42)	20.44 (15.82)	4.41 (3.95)	2.67 (1.96)	2.58 (2.77)	2.62 (2.97)	1.75 (1.89)	2.59 (1.90)	2.82 (3.11)
South	5.66 (4.53)	4.34 (4.20)	20.39 (13.97)	4.15 (3.41)	2.46 (1.84)	2.55 (2.42)	2.62 (2.69)	1.90 (1.74)	2.95 (1.88)	2.75 (3.18)
f	0.049	0.037	0.031	0.163	0.632	0.053	0.151	10.014	10.591	0.066
*p*	0.952	0.964	0.969	0.850	0.532	0.948	0.860	0.364	0.205	0.963
Home Conditions										
Lonely	7.95 (5.54)	5.71 (4.97)	23.40 (14.94)	4.67 (3.85)	2.60 (1.95)	3.17 (2.60)	4.86 (3.03)	1.85 (1.78)	2.64 (2.00)	3.62 (2.94)
In company	5.16 (4.35)	4.13 (4.13)	19.61 (14.80)	4.28 (3.73)	2.47 (1.88)	2.49 (2.63)	3.45 (3.75)	1.69 (1.70)	2.67 (1.99)	2.55 (3.02)
*t*	4.847	2.918	2.039	0.827	0.565	2.058	4.003	0.707	−0.118	2.869
*p*	0.000 ***	0.004 **	0.042 *	0.409	0.572	0.042 *	0.000 ***	0.480	0.906	0.005 **
d	0.609	0.468	0.456	0.103	0.069	0.260	0.513	0.110	0.015	0.528

*t* is the Student’s *t*-test value; *p* is the significance coefficient of the *t*-test; d is the Cohen’s d value; f is the value of ANOVA test. Bold values are all significant for * *p* < 0.05, ** *p* < 0.01, and *** *p* < 0.001.

**Table 5:** Differences between Groups (Student’s *t*-Test, ANOVA One Way). Psychological Variables: Resilience; COVID-19 Risk Factors: Emotional (Working Risk, Working Difficulties), and Fear of COVID-19 (COVID-19 Beliefs, COVID-19 Consequences).

Groups	*n* (%)	Resilience Mean (SD)	COVID-19 Emotional Risk Mean (SD)	COVID-19 Working Difficulties Mean (SD)	COVID-19 Beliefs Mean (SD)	COVID-19 Consequences Mean (SD)
Total Sample	425(100%)	55.45(8.32)			10.23(3.01)	12.28(1.86)
Gender						
Men	183 (44.1)	55.48 (8.35)	6.24 (2.35)	6.40 (2.32)	5.80 (2.80)	5.67 (2.29)
Women	242 (55.9)	55.42 (8.32)	7.22 (2.42)	7.13 (2.45)	6.56 (3.13)	6.10 (2.25)
*t*	-	0.073	−4.203	−3.116	−2.646	−1.898
*p*	-	00.942	0.000 ***	0.002 **	0.008 ***	00.058
d	-	0.007	0.513	0.305	0.371	0.190
AGE						
<40	192 (45.2)	54.27 (8.90)	6.87 (2.55)	6.70 (2.54)	6.51 (2.96)	5.48 (2.01)
≥40	233 (54.8)	56.42 (7.70)	6.74 (2.34)	6.91 (2.32)	6.01 (3.04)	6.27 (2.42)
*t*	-	−2.764	0.534	−0.892	1.698	−3.563
*p*	-	0.008 **	0.593	0.377	0.090	0.000 ***
d	-	**0.358**	0.052	0.086	00.167	**0.428**
Workplace Setting						
Hospital	246 (57.9)	55.48 (8.36)	6.78 (2.37)	6.60 (2.45)	6.12 (2.88)	5.87 (2.30)
Ambulatorial	179 (42.1)	55.41 (8.30)	6.82 (2.57)	7.12 (2.35)	6.39 (3.18)	5.97 (2.25)
*t*	-	0.083	−0.153	−2.229	−0.908	−0.457
*p*	-	0.934	0.879	0.026 *	0.364	0.648
d	-	0.008	0.042	0.304	0.093	0.044
COVID-19 Working Positions						
Directly involved	129 (30.4)	54.63 (8.53)	7.17 (2.58)	7.06 (2.37)	6.78 (3.11)	5.99 (2.08)
Not directly involved	295 (69.6)	55.69 (8.23)	6.64 (2.36)	6.72 (2.45)	6.00 (2.94)	5.86 (2.35)
*t*	-	−1.326	2.601	1.370	2.448	0.558
*p*	-	0.186	0.041 *	0.172	0.015 *	0.577
d	-	0.127	0.277	0.140	**0.260**	0.058
Previous Infection with SARS-CoV2						
Yes	57 (15.1)	54.40 (8.47)	7.23 (2.83)	6.36 (2.70)	6.68 (3.25)	5.51 (2.43)
No	368 (84.9)	55.58 (8.31)	6.75 (2.38)	6.88 (2.39)	6.18 (2.98)	5.96 (2.26)
*t*	-	−0.910	1.293	−1.371	1.010	−1.283
*p*	-	0.363	0.197	0.171	0.317	0.200
d	-	0.140	0.183	0.204	0.166	0.198
SARS-CoV2 infection of at least one Family Member or Friend						
Yes	299 (70.4)	55.35 (8.19)	6.89 (2.45)	6.82 (2.39)	6.35 (2.89)	5.83 (2.17)
No	126 (29.6)	55.678.75 ()	6.59 (2.41)	6.62 (2.50)	5.94 (3.28)	6.11 (2.50)
*t*	-	−0.353	1.174	0.008	1.282	−1.163
*p*	-	0.725	0.241	0.994	0.201	0.246
d	-	0.037	0.123	0.011	0.137	0.123
Preexisting Medical Conditions						
Yes	98 (23.1)	55.56 (8.79)	7.12 (2.25)	6.99 (2.54)	6.04 (3.31)	6.51 (2.71)
No	327 (76.9)	55.41 (8.19)	6.70 (2.49)	6.77 (2.39)	6.29 (2.92)	5.73 (2.10)
*t*	-	−0.155	−1.575	−0.795	0.719	−2.983
*p*	-	0.877	0.117	0.444	0.473	0.003 **
d	-	0.017	0.177	0.092	0.083	0.345
Italy Regions						
North	221 (52.0)	55.40 (8.58)	6.86 (2.48)	6.57	6.41 (3.10)	5.86 (2.40)
Center	97 (22.8)	56.36 (7.18)	6.80 (2.39)	6.95	6.16 (2.80)	5.74 (1.99)
South	106 (24.9)	54.74 (8.78)	6.66 (2.32)	7.24	5.92 (3.02)	6.15 (2.27)
f	-	0.972	0.248	20.927	0.981	0.896
*p*	-	0.379	0.780	0.037 *	0.376	0.409
Home Conditions						
Lonely	78 (18.4)	52.24 (9.27)	6.74 (2.47)	6.42 (2.35)	6.41 (2.78)	5.72 (2.20)
In company	346 (81.4)	56.15 (7.24)	6.81 (2.44)	6.92 (2.43)	6.21 (3.05)	5.96 (2.30)
*t*	-	−3.801	−0.223	−1.670	0.560	−0.867
*p*	-	0.000 ***	0.823	0.094	0.577	0.387
d	-	0.579	0.029	0.209	0.067	0.106

*t* is the Student’s *t*-test value; *p* is the significance coefficient of the *t*-test; d is the Cohen’s d value; f is the value of ANOVA test. Bold values are all significant for * *p* < 0.05, ** *p* < 0.01, and *** *p* < 0.001.

**Table 6:** Regression analysis.

Prediction Models	*R^2^*	*Adj. R^2^*	SE	*R^2^* Change	*F* Change	*P* *F* Change	*F*	*p*
Model A Predicted Variable—Depression								
Step 1	0.055	0.046	4.606	0.055	6.134	0.000 ***	6.134	0.000 ***
Step2	0.281	0.264	4.047	0.226	21.580	0.000 ***	16.127	0.000 ***
Step3	0.313	0.290	3.975	0.032	4.768	0.001 **	13.303	0.000 ***
Step4	0.462	0.442	3.523	0.149	112.479	0.000 ***	23.307	0.000 ***
Model B Predicted Variable—Anxiety								
Step 1	0.070	0.061	4.203	0.070	7.870	0.000 ***	7.870	0.000 ***
Step 2	0.250	0.231	3.803	0.180	16.434	0.000 ***	13.706	0.000 ***
Step 3	0.286	0.261	3.729	0.036	5.154	0.000 ***	11.657	0.000 ***
Step 4	0.398	0.376	3.428	0.112	75.758	0.000 ***	17.924	0.000 ***
Model C Predicted Variable—COVID-19 PTSD								
Step 1	0.058	0.049	14.526	0.058	6.426	0.000 ***	6.426	0.000 ***
Step 2	0.350	0.334	12.154	0.292	30.849	0.000 ***	22.181	0.000 ***
Step3	0.416	0.396	11.574	0.066	11.584	0.000 ***	20.781	0.000 ***
Step4	0.474	0.455	10.996	0.058	44.971	0.000 ***	24.484	0.000 ***

Bold values are all significant for ** *p* < 0.01 and *** *p* < 0.001.

**Table 7:** Regression analysis.

	*b*	*SE*	*β*	*t*	*p*
Model A—Depression (Dependent Variable)					
Predictors					
Age	−0.040	0.017	**−0.107**	−2.413	0.016 *
Gender	0.875	0.369	**0.092**	2.373	0.018 *
Civile Status	−0.353	0.422	−0.037	−0.835	0.404
Preexistent Medical Conditions	0.730	0.423	0.065	1.726	0.085
Home Conditions	−1.615	0.501	**−0.132**	−3.221	0.001 ***
COVID-19 Highly Impacted Region	0.031	0.362	0.003	0.084	0.933
Previous Infection with SARS-CoV2	−2.052	0.575	**−0.137**	−3.568	0.000 ***
SARS-CoV2 Infection of at least one Family Member or Friend	0.587	0.388	0.057	1.513	0.131
Beliefs on COVID-19 Contagion	0.331	0.070	**0.211**	4.718	0.000 ***
Consequences of COVID-19 Contagion	0.134	0.085	0.065	1.572	0.117
COVID-19 Emotional Risk	0.100	0.088	0.052	1.136	0.256
COVID-19 Working Difficulties	0.306	0.084	**0.157**	3.626	0.000 ***
COVID-19 Working Position	0.319	0.386	0.031	0.827	0.409
Workplace Setting	0.360	0.378	0.038	0.953	0.341
Resilience	−0.233	0.022	**−0.411**	−10.606	0.000 ***
Model B—Anxiety (Dependent Variable)					
Predictors					
Age	−0.061	0.016	**−0.176**	−3.729	0.000 ***
Gender	0.750	0.359	**0.086**	2.092	0.037 *
Civil Status	−0.087	0.411	−0.010	−0.213	0.832
Preexistent Medical Conditions	0.728	0.412	0.071	1.769	0.078
Home Conditions	−0.933	0.488	−0.083	−1.912	0.057
COVID-19 Highly Impacted Region	−0.055	0.352	−0.006	−0.155	0.877
Previous Infection with SARS-CoV2	−0.593	0.560	−0.043	−1.060	0.290
SARS-CoV2 Infection of at least one Family Member or Friend	0.137	0.377	0.014	0.364	0.716
Beliefs on COVID-19 Contagion	0.274	0.068	**0.190**	4.014	0.000 ***
Consequences of COVID-19 Contagion	0.181	0.083	**0.096**	2.186	0.029 *
COVID-19 Emotional Risk	0.146	0.086	0.082	1.699	0.090
COVID-19 Working Difficulties	0.257	0.082	**0.144**	3.136	0.002 **
COVID-19 Working Position	0.477	0.375	0.051	1.270	0.205
Workplace Setting	0.578	0.368	0.066	1.572	0.117
Resilience	−0.186	0.021	**−0.357**	−8.704	0.000 ***
Model C—COVID-19 PTSD (Dependent variable)					
Predictor					
Age	−0.200	0.052	**−0.168**	−3.821	0.000 ***
Gender	1.407	1.151	0.047	1.223	0.222
Civile Status	−0.252	1.318	−0.008	−0.191	0.849
Preexistent Medical Conditions	0.302	1.321	0.009	0.229	0.819
Home Conditions	−2.068	1.565	−0.054	−1.321	0.187
COVID-19 Highly Impacted Region	0.172	1.131	0.006	0.152	0.879
Previous Infection with SARS-CoV2	−1.327	1.795	−0.028	−0.739	0.460
SARS-CoV2 Infection of at least one Family Member or Friend	−1.283	1.211	−0.039	−1.059	0.290
Beliefs on COVID-19 Contagion	1.391	0.219	**0.281**	6.349	0.000 ***
Consequences of COVID-19 Contagion	1.033	0.266	**0.158**	3.879	0.000 ***
COVID-19 Emotional Risk	0.499	0.275	0.082	1.813	0.071
COVID-19 Working Difficulties	1.413	0.263	**0.230**	5.371	0.000 ***
COVID-19 Working Position	0.387	1.204	0.012	0.321	0.748
Workplace Setting	1.991	1.180	0.066	1.687	0.092
Resilience	−0.459	.068	**−0.257**	−6.706	0.000 ***

Bold values are all significant for * *p* < 0.05, ** *p* < 0.01 and *** *p* < 0.001.

## Data Availability

The datasets analyzed during the current study are available from the corresponding author on reasonable request.
